# The gut-brain axis and Parkinson disease: clinical and pathogenetic relevance

**DOI:** 10.1080/07853890.2021.1890330

**Published:** 2021-04-16

**Authors:** Elisa Menozzi, Jane Macnaughtan, Anthony H. V. Schapira

**Affiliations:** a Department of Clinical and Movement Neurosciences, UCL Queen Square Institute of Neurology, London, UK; b Institute for Liver and Digestive Health, University College London, Royal Free Campus, London, UK

**Keywords:** Parkinson’s, α-synuclein, gut-brain axis, microbiome

## Abstract

Gastrointestinal disorders are one of the most significant non-motor problems affecting people with Parkinson disease (PD). Pathogenetically, the gastrointestinal tract has been proposed to be the initial site of pathological changes in PD. Intestinal inflammation and alterations in the gut microbiota may contribute to initiation and progression of pathology in PD. However, the mechanisms underlying this “gut-brain” axis in PD remain unclear. PD patients can display a large variety of gastrointestinal symptoms, leading to reduced quality of life and psychological distress. Gastrointestinal disorders can also limit patients’ response to medications, and consequently negatively impact on neurological outcomes. Despite an increasing research focus, gastrointestinal disorders in PD remain poorly understood and their clinical management often suboptimal. This review summarises our understanding of the relevance of the “gut-brain” axis to the pathogenesis of PD, discusses the impact of gastrointestinal disorders in patients with PD, and provides clinicians with practical guidance to their management.

## 1. Introduction

Parkinson disease (PD) is the second most common neurodegenerative disease, affecting 1% of the global population over 60 years of age [1]. The pathological hallmarks of PD are progressive loss of nigrostriatal dopaminergic innervation in the substantia nigra pars compacta (SNpc) and presence of abnormal α-synuclein aggregates in the form of Lewy bodies (LBs) and neurites in different brain regions [2]. As a result of the striatal dopaminergic loss, patients with PD develop motor features such as bradykinesia, rigidity, tremor, and posture and gait impairment, and a plethora of nonmotor features, including manifestations of gastrointestinal (GI) dysfunction [3]. PD-related GI disorders represent a major source of functional disability and reduced quality of life, and are important determinants of treatment efficacy, making them central considerations in the clinical care of PD patients. Furthermore, mounting evidence suggests that pathological changes in the GI tract may precede alterations in the central nervous system, and that gut inflammation and changes in the intestinal microbiota may play a central role in PD pathogenesis [4].

This review examines the relationship between GI disorders and PD. We first summarise the pathogenetic role of the GI tract and the principles supporting the existence of a “gut-brain” axis in PD. From this perspective, we then discuss the most relevant PD-related GI disorders, focussing on practical implications of their management on the neurological function.

## 2. Where does Parkinson disease begin: the “Gut-Brain” theory

### 2.1. Principles and origin of the “Gut-Brain” axis

In 2003, Braak and colleagues put forth the “gut-brain” hypothesis according to which PD may be caused by an unknown neurotropic pathogen that could pass the mucosal barrier of the GI tract, cause pathological α-synuclein aggregation in the enteric nervous system (ENS), and then spread by retrograde axonal transport through the vagus nerve (VN) to the dorsal motor nucleus of the vagus (DMV) and the brain [5]. Neuropathological findings of LBs in the ENS and the DMV of individuals with PD were the first evidence supporting this theory [6]. The presence of gastric α-synuclein immunoreactive inclusions in both the myenteric Auerbach and the submucosal Meissner plexuses of five people showing LBs pathology in the brain further reinforced this idea [7]. Subsequently, the evidence that misfolded α-synuclein could act as a template, triggering further α-synuclein to misfold with formation of insoluble inclusions capable of spreading from neuron to neuron [8,9], provided a mechanistic basis for Braak’s hypothesis.

The evaluation of α-synuclein aggregates in the GI tract of patients in the prodromal or manifest phase of PD has been the focus of several research groups over the past 20 years, but conflicting data have been reported so far. Phosphorylated α-synuclein deposition has been observed in the submucosal nerve fibres or ganglia of subjects with idiopathic rapid eye movement (REM) sleep behaviour disorder (iRBD), a prodromal marker of PD [10], as well as in the ENS of gastric, duodenal and colonic biopsies undertaken by PD patients years before the presentation of motor signs [11–13]. These findings may therefore support the presence of pathogenetic changes in the prodromal phase of PD. However, there is still a relative lack of cases showing isolated α-synuclein pathology in the GI tract [14]. Furthermore, similar α-synuclein or phosphorylated-α-synuclein immunoreactivity was reported comparing intestinal samples of PD patients and healthy age-matched controls [15,16], although the application of quantitative morphometry could detect differences in the amount and pattern of phosphorylated-α-synuclein aggregates between the two groups [16]. Several factors may explain the variability of these pathological findings, e.g. lack of standardised immunohistochemical protocols, different GI tract sites examined, and wide and heterogenous distribution of the peripheral nervous branches [17]. The application of new functional imaging technologies could provide a unique, non-invasive opportunity to prove the relevance of the gut-brain axis *in vivo* and therefore resolve some of these discrepancies [18]. The quantification of acetylcholinesterase density in peripheral organs by using the PET tracer [^11^C]-donepezil is a validated method to assess the parasympathetic gut innervation [18]. Studies conducted on early-stage PD patients found a [^11^C]-donepezil signal decrease in both the colon and small intestine [19], and similar findings were replicated in a cohort of patients with iRBD, suggesting that the cholinergic innervation of the intestine is impaired during the prodromal phase of PD [20]. These findings have been replicated in a cohort of *de novo* PD patients, with and without prodromal RBD (i.e. prior to parkinsonism). PD patients with prodromal RBD had reduced ^123^I-metaiodobenzylguanidin (MIBG) heart:mediastinum ratios, reduced colon [^11^C]-donepezil uptake values, enlarged colon volumes and delayed colonic transit times compared to PD patients without RBD, with similar reduction in putaminal ^18^F-dihydroxyphenylalanine (FDOPA) PET uptake [21]. Interestingly, PD patients with RBD and patients with iRBD showed similar MIBG and donepezil PET scans [21]. On the one hand, these findings suggest that there is a subset of PD patients (the ones without prodromal RBD) who do not fit within the “gut-brain” theory as they are characterised by primary loss of putaminal FDOPA uptake with secondary sympathetic and parasympathetic denervation (thus following a “brain-first” trajectory), but on the other hand they do confirm the existence of a “gut-first” subtype of PD patients. In these patients, α-synuclein pathology might start within the enteric and parasympathetic nervous system, leading to prodromal GI disorders such as constipation, and then propagate in the sympathetic nervous system and lower brainstem, leading to prodromal RBD [21].

### 2.2. The vagus nerve is a possible route of retrograde transport of α-synuclein from the gut to the brain

If α-synucleinopathy first appears in the gut, how does it reach the central nervous system? It has been hypothesised that the VN is a potential pathway of retrograde transport of α-synuclein between the ENS and the brain. In rats, vagal efferent axons and terminals originating from the DMV express α-synuclein and some of these preganglionic efferents form synapses with α-synuclein-positive intrinsic neurons in the myenteric plexus of both the stomach and duodenum [22]. Animal models of PD have confirmed the involvement of the VN. In fact, when low doses of rotenone were chronically injected in the stomach of mice, α-synuclein increased in the ENS and subsequently spread to the DMV and SNpc, with selective dopaminergic cell loss [23]. These pathological effects and the development of motor deficits were partially prevented when mice were pre-treated with hemi-vagotomy [24]. Similar results supporting a caudo-rostral spread were also confirmed in other experiments evaluating the effects of the injection of recombinant adeno-associated viral vectors (AAV) expressing α-synuclein in the VN of the rat neck [25], or pathological forms of α-synuclein in the gut [26]. In the latter experiments, the injection of pathological α-synuclein obtained from PD brain lysate and distinct recombinant α-synuclein forms in the intestinal wall of rats, resulted in spread of α-synuclein to the VN, DMV and brainstem in a time-dependent manner [26]. In another study, the inoculation of α-synuclein pre-formed fibrils (PFF) into the mouse GI tract induced LB-like aggregates in the DMV but no further caudo-rostral propagation beyond [27]. The further propagation of pathological α-synuclein up to the lower brainstem and onto the SNpc, was reported after α-synuclein PFF injection into the duodenum and pyloric muscle layer of mice, together with dopaminergic cell loss and development of motor deficits [28]; truncal vagotomy prevented the caudo-rostral spread of α-synucleinopathy and the onset of behavioural deficits [28]. In another mouse model, duodenal α-synuclein PFF inoculation was capable of disrupting GI function, increasing phosphorylated α-synuclein in myenteric neurons, and in aged mice, increasing brainstem phosphorylated α-synuclein and decreasing striatal dopamine [29].

Indirect evidence supporting a central role of the VN in PD pathogenesis comes from large population-based studies where the risk of developing PD has been assessed in people who underwent vagotomy versus matched controls (for a comprehensive review of this topic, the reader is directed elsewhere [30]). Essentially, these studies have shown that vagotomy, especially truncal vagotomy, might reduce the risk of developing PD > 5 years after the procedure [31,32]. A possible explanation why truncal rather than selective vagotomy could reduce the risk of PD might be that the vermiform appendix is the first locus of enteric α-synuclein aggregation, due to its abundant content of α-synuclein [33], the lack of blood-tissue barrier in the mucosa – which makes it more susceptible to environmental triggers [30], and the connection with vagal efferents [34]. Indeed, some observational and population-based studies have reported that appendectomy delayed PD onset [35,36], and even reduced the risk of developing PD by 19.3% [35]. However, two large-scale cohort studies did not confirm these findings, showing a higher incidence of PD in subjects shortly after appendectomy or with long-term follow up after the procedure [37,38]. Overall, these results may support a protective role of vagotomy, whereas an association between appendectomy and reduced risk of PD is less clear.

### 2.3. The “leaky gut” and enteric immune dysregulation contribute to PD pathogenesis

The intestinal epithelial barrier is supposed to separate the pro-inflammatory contents of the intestine from the systemic circulation, however if “leaky”, it may allow factors, such as bacterial lipopolysaccharide (LPS), to enter the gut lumen and reach the ENS. Several lines of evidence support the presence of increased colonic (but not small intestine) barrier permeability in PD patients [39].

Urinary excretion of orally ingested sugar is typically used to evaluate the barrier function of different intestinal sites. Alterations in mannitol and lactulose excretion reflect changes in permeability of the small intestine, whereas the addition of either sucralose or chromium-labeled EDTA is used to evaluate changes in colonic permeability [39]. In small cohorts of PD patients versus control subjects, significantly higher 24 h urinary excretion of sucralose was observed [40], but no differences in urinary mannitol and lactulose excretion [41], suggesting an increased permeability of the colon only.

Measurement of alpha-1-antitrypsin and zonulin in the faeces is another method to evaluate intestinal barrier *in vivo*. Zonulin is a tight junction-associated cytoplasmic protein, whose faecal increase is a marker of reduced integrity of the intestinal barrier [39]. Alpha-1-antitrypsin is a protease inhibitor, whose faecal detection is equally considered a measure of mucosal barrier integrity because it reflects loss of proteins to the intestinal lumen [39]. In a case-control study conducted on PD patients and age-matched controls, faecal alpha-1-antitrypsin and zonulin were significantly elevated in PD patients [42]. Although of interest, all these findings do not discriminate between a perturbation of the mucosal barrier due to damage to and loss of epithelial cells, or due to dysfunctional tight junctions [39]. To tackle the issue, tight junction proteins expression has been analysed in colonic biopsies of PD patients, finding significantly lower occludin, but not zonulin, and aberrant subcellular distribution of tight junction proteins [43], or reduced zonulin [40], in PD as compared to controls.

A possible role of intestinal inflammation in PD pathogenesis is also supported by the association between inflammatory bowel disease (IBD) and PD. A recent meta-analysis showed that the overall risk of PD in IBD is significantly higher than in controls (RR 1.41, 95% CI 1.19–1.66), with Crohn’s disease having an increased 28% and ulcerative colitis an increased 30% risk of PD, respectively [44]. The presence of shared alleles in the leucine-rich repeat kinase 2 gene (*LRRK2*, OMIM 609007) in both PD and Crohn’s disease [45] further reinforces the hypothesis that the two diseases might share pathogenetic pathways involving the dysregulation of the intestinal barrier/immune system. Interestingly, IBD patients receiving anti-tumor necrosis factor therapy had a 78% reduction in the risk of developing PD compared with patients who did not (adjusted incidence rate ratio, 0.22; 95% CI, 0.05–0.88) [46], another finding favouring a potential role for systemic inflammation in PD pathogenesis.

### 2.4. The perturbation of the gut microbiome as potential trigger of PD

The attention drawn to the GI tract as initial site of α-synucleinopathy, has raised much interest around the potential causative role of the gut microbiome in triggering the pathological changes seen in PD. Evidence from animal studies seems to support this view. In a transgenic mouse model of PD that overexpresses α-synuclein, the presence of gut microbes could exacerbate motor and GI deficits, microglial activation and brain α-synuclein pathology, whereas antibiotic treatment reduced PD-related motor deficits [47]. Moreover, when specific microbial metabolites were orally administered to germ-free mice, they were capable of promoting neuroinflammation and motor symptoms, and the colonisation of these mice with the microbiota transplanted from PD patients was able to increase motor dysfunction compared to transplant from healthy human donors [47]. Furthermore, faecal transplantation from 1-methyl-4-phenyl-1,2,3,6-tetrahydropyridine (MPTP)-treated mice to normal mice led to impairment of motor functions and decline in the striatal dopamine and serotonin levels, whereas transplantation of faecal microbiota from normal mice to MPTP mice reduced motor dysfunction, rescued striatal dopamine and serotonin levels and reduced nigral activation of microglia and astrocytes [48].

What is the evidence in humans? Despite the several limitations related to the design of studies of the human microbiome (differences in geographical background, age, sex, diet, medications, occurrence of gastrointestinal symptoms influencing the gut microbiome composition), there are several reports that the intestinal microbiome of PD patients is different in comparison to that of healthy individuals [49]. The main findings include a decreased abundance of genus *Prevotella*, *Lachnospiraceae* family, including the short-chain fatty acids-producing genera *Blautia* and *Roseburia*, and genus *Faecalibacterium*, and an enrichment in *Lactobacillaceae* family, and genera *Akkermansia* and *Bifidiobacterium* [49,50]. The main question that these studies did not address is whether these changes are cause or consequence of the disease [51]. Preliminary findings from studies evaluating the composition of the gut microbiome of patients with iRBD or elderly people, suggest that some changes in the microbiome composition might indeed precede the development of parkinsonism and therefore represent important prodromal biomarkers of PD [52,53].

If these findings are confirmed in larger cohorts of individuals at risk of developing PD, the question about how gut microbes are detected in the GI tract and trigger α-synucleinopathy, remains to be answered. Some have proposed a potential role of enteroendocrine cells in this process. Enteroendocrine cells are specialised intestinal cells present in the small intestine and colon, which are capable of sensing ingested nutrients and bacterial by-products [54]. Their membranes contain voltage-gated ion channels making them electrically excitable [55], and they have neuronal-like properties, including synaptic features [56]. The recent discovery that enteroendocrine cells can express α-synuclein [57] and synapse with the VN forming a neuroepithelial circuit that connects the gut lumen to the brainstem [58], might let us speculate that once stimulated by gut bacteria signalling, these cells may trigger α-synucleinopathy within the ENS, and subsequently spread it through the VN to the brain. The increased gut wall permeability seen in patients with PD may contribute in helping bacterial by-products or toxins (e.g. LPS) to access the ENS, induce local inflammation and trigger synucleinopathy [59]. In addition, the leaky gut syndrome may also allow bacterial by-products to access the blood stream and increase systemic inflammation, as reflected by increased levels of pro-inflammatory cytokines in the serum of PD patients [60]. This pro-inflammatory status may contribute to the disruption of the blood-brain barrier (BBB), activation of microglial cells, and ultimately nigral dopaminergic cell death [61]. Future studies testing these hypotheses will hopefully provide insight into pathogenetic mechanisms underlying PD.

## 3. PD-related GI disorders and the gut-brain axis: practical implications

**Figure 1.**
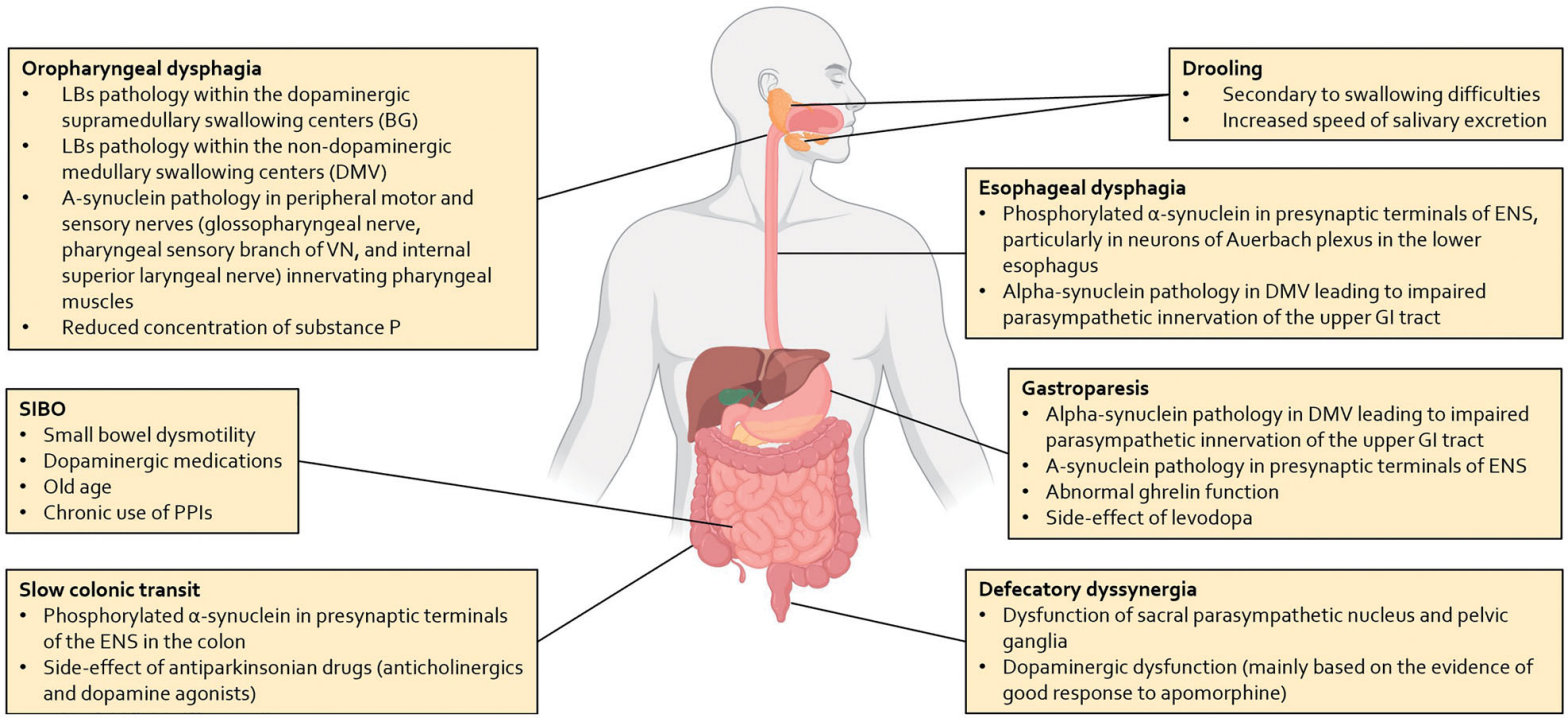
Summary of pathophysiological mechanisms associated with the gastrointestinal disturbances in patients with PD. The pathophysiology of these symptoms is complex and involves both central and peripheral mechanisms. LBs: Lewy bodies; BG: basal ganglia; DMV: dorsal motor nucleus of the vagus; GI: gastrointestinal; VN: vagus nerve; SIBO: small intestinal bacterial overgrowth; PPIs: proton-pump inhibitors; ENS: enteric nervous system.

Disorders of the GI tract are one of the most frequent and disturbing symptoms affecting patients with PD [3], and ultimately an important cause of disability, reduced quality of life and hospitalisation [62]. The entire GI tract can be affected in PD, with a variety of disorders including dental problems, drooling of saliva, oro-pharyngeal/esophageal dysphagia, gastroparesis, and constipation [63]. These manifestations mainly arise from an early and extensive involvement of the peripheral components of the autonomic nervous system, including the ENS and the sympathetic and parasympathetic ganglionic neurons and postganglionic projections, and to a lesser extent, of the central autonomic network, in particular the DMV and the rostral ventrolateral medulla (see Figure 1) [64].

The epidemiological, clinical, and therapeutic features of PD-related GI disorders are summarised in Table 1 (for a more extensive description of these conditions, the reader is directed elsewhere [63,65]). Hereafter, we wish to present the most relevant PD-related GI disorders within the context of the gut-brain axis. Driven by a practical approach, we will discuss how GI disorders (the *gut* level) can impact neurological function (the *brain* level) in PD, and how their manipulation can affect neurological outcomes.

**Table 1.** Clinical, diagnostic and therapeutic features of the main GI disturbances in PD.

GI disturbance	Prevalence	Diagnosis	Non-pharmacological management	Pharmacological treatment	Surgical treatment
Sialorrhea (or drooling of saliva)	10–81%		Chew gum, rehabilitation of deglutition	Oral glycopyrrolate, sublingual atropine drops, botulinum toxin injections into salivary glands	
Oropharyngeal dysphagia	35–80%	FEES, barium swallow studies, videofluoroscopy, HRM	Postural changes, reduced meal volumes, eating slowing, use of liquid thickeners, EMST, VAST	Levodopa (?)	DBS (?)
Esophageal dysphagia	∼ 30%	HRM with or without fluoroscopy	Same as oropharyngeal dysphagia	Botulinum toxin injections in the distal esophageus; Levodopa (?)	STN DBS (?); pneumatic dilation or surgical therapies for EGJ outflow obstruction
Delayed gastric emptying (gastroparesis)	70–100% (symptoms are reported only in 25–45% of cases)	Gastric emptying scintigraphy; wireless motility capsule	Eating small, frequent meals with low fibre and low-fat content	Domperidone; highly selective 5-HT4 agonists (prucalopride, mosapride – not approved in the United States); botulinum toxin injections into the pyloric sphincter; ghrelin agonist (?)	Gastric pacemaker implantation (?)
SIBO	25–54%	Lactulose or glucose breath tests	Probiotics	Antibiotics; correction of SIBO causes (e.g., prokinetic drugs)	
Constipation	∼ 50% (range, 8–70%)	Colonic transit using radio opaque markers, MRI defecography, HRAM	Increasing high fibre dietary and fluid intake, performing physical activity, removing aggravating medications, probiotics and prebiotic fibre. For defecatory dyssynergia: biofeedback therapy	Oral bulking agents, osmotic laxatives (macrogol), lubiprostone. For defecatory dyssynergia: levodopa, apomorphine, botulinum toxin injections into the external anal sphincter and puborectalis	

EGJ: esophagogastric junction; EMST: expiratory muscle strength training; FEES: flexible endoscopic evaluation of swallowing; HRAM: high-resolution anorectal manometry; HRM: high-resolution manometry; SIBO: small intestinal bacterial overgrowth; STN DBS: subthalamic nucleus deep brain stimulation; VAST: video-assisted swallowing therapy; (?): contrasting or insufficient results that require further confirmation.

### 3.1. Delayed gastric emptying

Delayed gastric emptying (DGE) is defined as reduced stomach motility in the absence of mechanical obstruction, and when symptomatic it is referred as gastroparesis [66]. Although only 15–45% of PD patients report symptoms suggestive of gastroparesis (such as nausea, vomiting, postprandial bloating, early satiety, or abdominal pain) [67,68], DGE is found in 70–100% of PD patients [69], and can occur in both early and advanced stages of PD [66]. The pathophysiology of this phenomenon is complex and likely multifactorial. The damage to, and α-synuclein deposition in the VN and ENS likely play a major role in DGE, whereas more controversial is the involvement of Cajal interstitial cells (the gastric pacemaker cells) [70,71]. Common medications used in PD, such as levodopa and anticholinergics, can contribute to DGE in a dose-dependent manner [71,72], and the interaction of some macronutrients (especially fats) with receptors in the small intestine can further inhibit gastric emptying [73].

Since levodopa absorption takes place only in the proximal duodenum, DGE is the rate-limiting step in this process [66]. In one study conducted on 31 PD patients, peak-time gastric emptying rates correlated with plasma levodopa peak delay levels, suggesting a direct relationship between DGE and “delayed on-time” [74]. In another study comparing PD patients with and without motor fluctuations, the burden of motor fluctuations was associated with more severe DGE rates [75]. It is worth remembering that DGE is only one of the several factors and/or conditions related to the GI tract that can influence levodopa bioavailability, and others will be discussed in the dedicated section below. Among these, diet is a major determinant due to competition between levodopa and dietary large neutral amino acids (LNAA, such as phenylalanine, tyrosine, and tryptophan) for specific active-transport systems in the small intestine and at the BBB, with high-protein dietary regimes associated with suboptimal motor response [76]. Given these premises, it is clear that recognising and treating PD-related gastroparesis is of paramount importance, not only to reduce the gastrointestinal symptoms associated with this condition, but also to ameliorate levodopa bioavailability and consequently motor function.

Dietary changes represent a first-line strategy to treat gastroparesis in PD. Adjusting fat intake in combination with high quality carbohydrates (starch), could improve the pattern of gastric emptying and thus levodopa bioavailability [76]. Levodopa absorption may also be benefitted by synchronising meal times in relation to oral levodopa dosing [77], such that patients are generally advised to take their medication about 30–45 min before eating [76]. In advanced PD stages, redistribution of daily protein intake (limited to evening hours) and/or restriction of daily protein intake, have been shown to increase levodopa bioavailability [78], reduce plasma levels of LNAA [78,79], improve motor fluctuations in 30–90% of PD patients [79–82] and reduce levodopa daily dose in 75% [78]. However, careful monitoring is necessary with long-term adherence to low-protein regimens, as these may increase the risk of nutritional complications such as weight loss and malnutrition [71]. Administration of dietary herb extract Rikkunshito has been evaluated in two small open-label trials. Contradictory results were found on DGE (improved in one study, no effect in the other), whereas effect on motor fluctuations was not evaluated [83,84].

Prokinetic agents are a mainstay treatment for gastroparesis [71]. Of all the available prokinetics, domperidone, a D2 dopamine receptor blocker that does not cross the BBB, has been shown to be efficacious in the management of gastroparesis in PD, without worsening motor symptoms [85], and it increases levodopa bioavailability when co-administered with levodopa [86]. The updated recommendations on PD treatment released by the International Parkinson and Movement Disorder Society (MDS) Evidence-Based Medicine (EBM) Committee, consider domperidone “possibly useful” in the treatment of PD-related gastroparesis but specialised monitoring is required due to potentially life-threatening arrythmias secondary to QT elongation [87]. Metoclopramide, the only FDA-approved medication for gastroparesis in the United States, is contraindicated in PD due to its ability to cross the BBB and block central dopaminergic receptors [71]. Other prokinetics which have been under investigation in PD include mosapride, a selective 5-hydroxytryptamine type 4 (5-HT_4_) agonist, which was beneficial at reducing gastric emptying rates, prolonging “on” time and reducing motor fluctuations in a small open-label trial on 5 patients with advanced PD [88]. Another prokinetic agent with potential application in PD-related gastroparesis is ghrelin. Ghrelin is a gastric-derived hormone that increases appetite and food intake and stimulates secretion of growth hormone [89]. Due to the ability to stimulate gastric emptying in humans [90], both ghrelin and ghrelin receptor agonists have been successfully applied in the treatment of gastroparesis associated with several diseases such as diabetes [91,92]. To date, studies evaluating ghrelin in PD patients with gastroparesis are lacking, however preclinical evidence has shown favourable effect of ghrelin [89] and ghrelin agonists [93,94] on reducing levodopa-induced DGE and increasing plasma levodopa levels. Finally, macrolides, by acting as motilin agonists, are potent prokinetics that have been proposed as a possible strategy to treat PD-related gastroparesis but for now clinical data are lacking to support their use in routine practice [95].

In summary, current management for PD-related gastroparesis is limited. Initial steps should include review of concomitant conditions/medications known to delay gastric emptying and dietary adjustments. Low-protein intake and/or protein restriction to evening hours, should be proposed to patients with advanced stages and troublesome motor fluctuations, but with cautious monitoring on possible long-term complications. The judicious use of domperidone, always combined with cardiac safety monitoring, may be beneficial in some patients. Future studies are needed to explore the potential role of 5-HT_4_ agonists, ghrelin, and macrolides.

### 3.2. Helicobacter pylori *infection*

Aside from its pathogenic role in the development of peptic ulcers and gastric cancer, chronic systemic inflammation secondary to *Helicobacter pylori* (HP) infection has been linked to the development of various neurological disorders, including PD [63]. HP infection increases the risk of developing PD by 1.5–3-fold [96,97], and HP-positive patients display worse motor function compared with HP-negative patients [98]. However, how HP infection may impact PD motor function, is a controversial subject, and multiple mechanisms have been postulated.

The most studied mechanism is the effect on levodopa bioavailability. Pierantozzi and colleagues reported increased levodopa concentration in the elimination phase after HP eradication [99], whereas other groups did not report any difference in levodopa pharmacokinetic parameters between HP-positive and HP-negative patients [100]. Other mechanisms leading to a deterioration in motor function in HP-positive patients might include production of neurotoxic factors, such as cholesterol glycosides, ultimately leading to degeneration of dopaminergic neurons in the brain, induction of local and systemic immune response leading to BBB disruption, neuroinflammation and loss of dopaminergic neurons, generation of autoantibodies against functional proteins in the brain, direct entrance of HP in the brain through intracellular transport, and dysregulation of the gut microbiota [101–103]. In addition, hypochlorhydria induced by HP could promote the development of small intestinal bacterial overgrowth (SIBO) [104], which is itself known to worsen motor function and fluctuations in PD (see below) [105].

Eradication therapy of HP might therefore represent a potential *gut*-level treatment to improve *brain*-level function in PD. Three randomized-controlled trials (RCT) of HP eradication in PD have been conducted so far, with contrasting findings. After HP eradication, two studies reported improvement in the “on” time (during a levodopa-challenge protocol) [99], and in the stride length [106]. In one of these studies, clinical improvement was coupled with significant increased levodopa absorption following HP eradication [99]. However, a recent single-center RCT conducted on 67 PD patients showed no improvement of motor outcomes after 12 weeks, and no changes in non-motor symptoms and quality of life at week 12 and 52 [107]. Interestingly, the latter study evaluated the role of concomitant SIBO, finding no significant improvement in motor outcomes after HP eradication in those patients who were SIBO negative at baseline, while conversion to SIBO negativity did not improve motor function in initially SIBO-positive patients [107]. Overall, these results seem to argue against the hypothesis that SIBO may play a concomitant role with HP infection in determining motor function in PD [107].

The available evidence is therefore too limited to justify HP eradication to improve PD motor symptoms, and for now HP screening should not be part of standard clinical care of PD [107]. Larger multicenter studies evaluating clinical and nonclinical outcomes (for instance, neuroinflammatory markers) are required to clarify whether a causal relationship between HP infection and PD does exist and disentangle the underlying mechanisms of such relationship.

### 3.3. Small intestinal bacterial overgrowth

Small intestinal bacterial overgrowth (SIBO) is a condition associated with increased concentration of bacteria (above 10^5^ colony forming unit/mL) and/or the presence of colonic-type bacteria in the small intestine [108,109]. Recent studies have shown an increased prevalence of SIBO in patients with PD (25–54%), and a strong association between SIBO and the occurrence of motor complications and fluctuations [105,110,111].

Unfortunately, our understanding of the causative role of SIBO in PD remains unclear. In previous studies, several factors (such as lack of standardised protocols for breath tests for SIBO [111], or spontaneous changes in SIBO status during the disease course [107,112]), have limited interpretation. SIBO-positive patients may display increased intestinal permeability that facilitates bacterial translocation, creates a proinflammatory environment, and ultimately promotes microglial activation and neurodegeneration (e.g. abnormal accumulation of α-synuclein in enteric neurons) [111]. Alternatively, SIBO might affect levodopa bioavailability, either as a result of peripheral inflammation or partial metabolism of levodopa [63]. Recent studies reporting the effect of small intestine microbiome on peripheral levodopa conversion, supports this last hypothesis. The first step of bacterial levodopa metabolism, the decarboxylation of levodopa to dopamine, is in fact carried out by bacterial tyrosine decarboxylase (tyrDC), which is encoded on genomes of several species in the small intestine [113]. Among all the identified bacterial species, only *Enterococcus faecalis* was able to completely metabolise levodopa, and the abundance of *Enterococcus faecalis* and tyrDC correlated with levodopa and dopamine metabolism in human gut microbiota samples [114].

It is therefore tempting to speculate that SIBO eradication might be a therapeutic option to improve motor function in PD. Eradication of SIBO has been tested in an open-label trial of rifaximin 400 mg three times a day for 1 week, with evidence of improved motor fluctuations (“off” time and “delayed on”) but no changes in levodopa pharmacokinetic parameters [105]. Another RCT of rifaximin in SIBO-positive PD patients with at least 4 h/day of “off” time, has been designed to evaluate “off” time reduction. Unfortunately, the results of this trial were inconclusive. According to the authors, the reasons behind this failure might be ascribed to recruitment difficulties and unexplained spontaneous conversion to SIBO negativity in patients treated with placebo [112], proving how challenging the design of studies evaluating SIBO eradication in PD can be.

Although the available evidence does support an association between SIBO and motor deterioration in PD, whether and by what antibiotic regimen SIBO should be eradicated remains to be established. A suggestion that some bacterial species of the small intestine such as *Enterococcus faecalis* and tyrDC enzymatic activity levels may serve as biomarkers for monitoring levodopa efficacy has been made, therefore the impact of tyrDC manipulation on levodopa efficacy in PD patients undoubtedly deserves further attention [114].

### 3.4. Constipation

Constipation, one of the most prevalent PD-related GI disorders, can precede the onset of motor manifestations by 20 years [115,116]. It correlates with duration and severity of PD [117], being observed more frequently in older patients with more advanced stages [118,119]. Constipation is associated with severe psychosocial distress [120] and serious complications including colonic volvulus, intestinal pseudo-obstruction, megacolon, and faecal impaction [118].

Lacking a unique definition, the prevalence of constipation varies across PD studies, ranging from 8% to 70% [121], and can be subjectively reported by patients as hard stools, reduced bowel movements, bloating, abdominal pain and straining during defaecation [122]. PD-related constipation may stem from either colonic slow transit or anorectal dysfunction, that can play a role alone or in combination [121]. The underlying mechanisms include cell death and α-synuclein accumulation in the parasympathetic and sympathetic neurons innervating the entire colon, and increased colonic inflammation and permeability, as discussed above [18]. Moreover, a direct association between PD-related constipation and changes in the gut microbiome might exist. Preclinical studies have demonstrated that constipation is reduced in germ-free α-synuclein transgenic mice compared with mice hosting a complex microbiota [47]. Results from clinical studies have also suggested that PD patients with constipation may display a distinctive gut microbiome signature [123,124]. In one study, several taxa including *Dorea*, *Oscillospira*, *Akkermansia*, and *Ruminococcaceae*, were positively associated with chronic constipation and stool consistency, whereas *Faecalibacterium* and butyrate producing-bacteria displayed a negative association [123]. Similar findings were replicated by another study where constipation increased family Lactobacillaceae, but decreased genus *Faecalibacterium* [124]. However, other groups were unable to demonstrate a relationship between specific gut microbiome profiles and constipation [125]. Despite some discrepancies among these results, modulating the *gut* microbiota may represent one the most exciting and powerful therapeutic options for constipation in PD.

Dietary changes, probiotics and prebiotic fibres, all represent potential strategies that may alleviate constipation by manipulating the gut microbiota [126]. Two studies have evaluated the effect of dietary insoluble fibres (dietetic supplements composed of 375 mg wheat, 70 mg pectin, 2.5 mg dimethylpolyoxyhexane-900 [127]; or psyllium [128]) in the treatment of PD-related constipation. Beneficial effects were reported on some constipation-related measures (severity, stool frequency or weight), although the small sample sizes of both studies (*n* = 19, and *n* = 7 patients) might have underpowered these findings [127,128]. Interestingly, motor function and plasma levodopa levels were ameliorated by insoluble fibre-enriched diet, both at 2 week- and 2 month-follow up [127]. More recently, the effect of probiotics on PD-associated constipation has been under investigation. Probiotics are live or attenuated microorganisms with multiple putative beneficial effects on intestinal homeostasis [129]. In several PD animal models, administration of probiotics could reduce motor impairment and exert neuroprotective effects on dopaminergic neurons [130,131]. To date, three studies, two of whom were RCT, have evaluated the use of probiotics in PD-related constipation. Consistent improvement in several constipation-related measures (spontaneous bowel movements, stool consistency), patients’ quality of life and treatment satisfaction has been reported following multi-strain probiotics treatment, alone or in combination with dietary fibres, or single-strain probiotic combined with dietetic therapy [132–134]. Based on these results, the updated recommendations by the MDS EBM Committee consider probiotics and prebiotic fibres as “clinically useful”, and their application in clinical practice an “acceptable risk without specialized monitoring” [87]. However, whether these treatments may positively impact other outcomes such as motor symptoms, has not been investigated yet.

The osmotic laxative macrogol [135] and chrolide type 2 channel activator lubiprostone [136] have been evaluated in two RCTs. Both treatments showed beneficial effects on constipation-related outcomes (stool frequency and consistency, gastrointestinal symptoms) but no changes in motor outcomes in the treatment group [135,136]. Currently, macrogol and lubiprostone are considered “possibly useful” for constipation in PD.

In summary, the clinical management of PD-related constipation is challenging, and fulfilling the ultimate goal of patients’ satisfaction is not achieved by currently available treatments [137]. We believe that a personalised approach combining non-pharmacological and pharmacological options might be the best option to consider. First-line treatment should include nonpharmacological measures such as lifestyle modifications (increase physical activity, increase amount of fluid and fibre intake up to 20–35 g/day), and removal of concomitant aggravating medications (e.g. opioids, amantadine) [118,138]. Although formal guidelines are currently lacking, probiotics and prebiotic fibres in the first instance, followed by lubiprostone and macrogol, could be considered at the individual level when previous strategies have failed.

## 4. Conclusions

**Figure 2.**
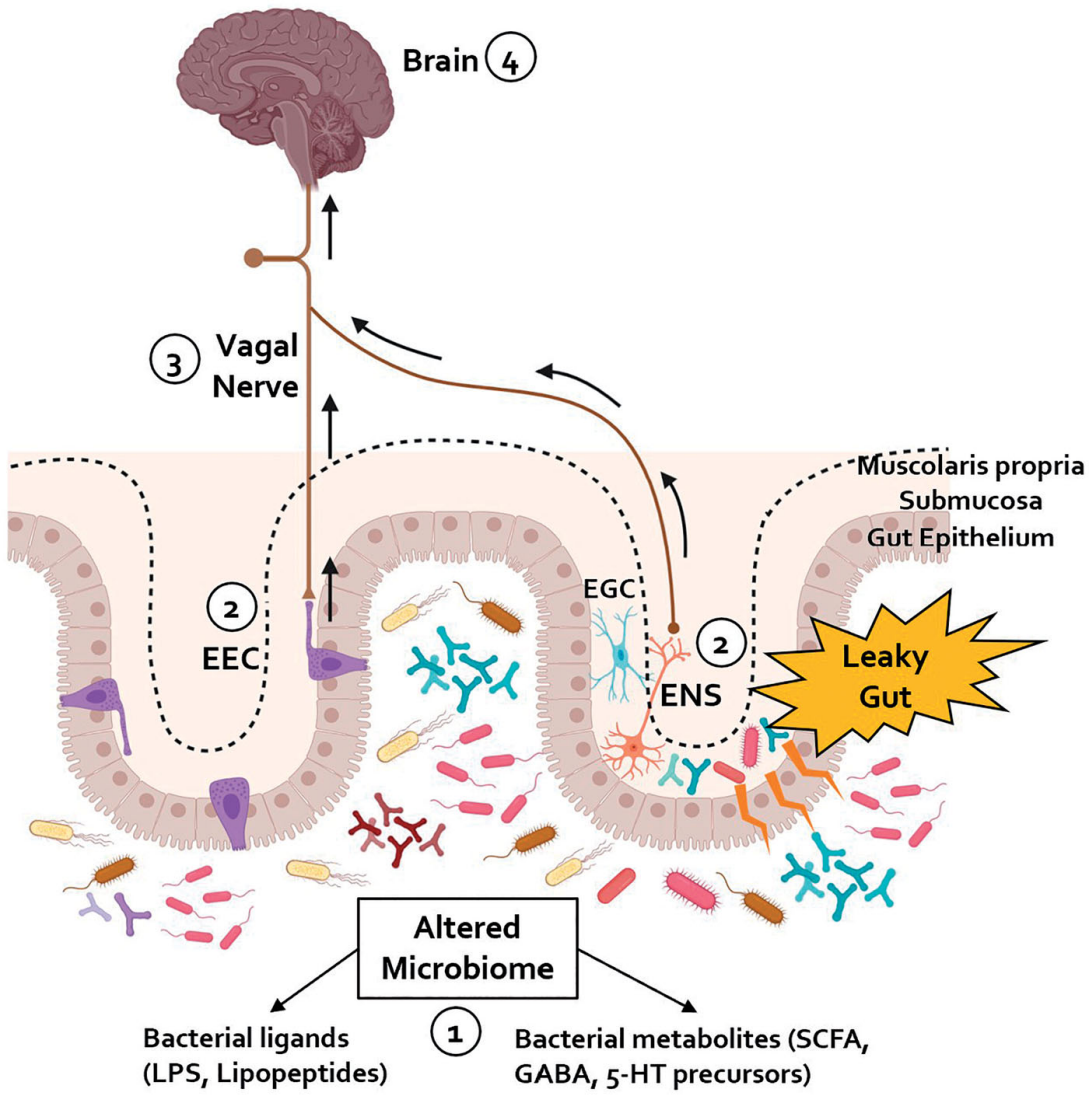
A proposed schematic representation of the pathways implicated in the gut-brain axis in PD. (1) An alteration in the composition of the gut microbiome towards a more pro-inflammatory profile has been shown in patients with PD. (2) The increased gut permeability seen in patients with PD could expose enteric neurons to bacterial derived pro-inflammatory products; the activation of enteric glial cells (EGC) within the gastrointestinal tract of PD patients, seen in the early phases of the disease, might contribute to amplify the impairment of the intestinal barrier and facilitate the spread of pathological α-synuclein within the ENS. Alternatively, bacterial products could be sensed by the EECs dispersed within the gut epithelium; these cells might be a site of initial α-synuclein aggregation which can then be transferred to the VN. (3) Either through the mediation of the ENS or by direct synapsing, pathological α-synuclein can reach the vagal nerve and, due to its cell-to-cell transmission properties, retrogradely propagate to the brain. (4) Within the brain, pathological α-synuclein spreads within different areas resulting in loss of nigrostriatal dopaminergic neurons and development of motor symptoms of PD. ENS: enteric nervous system; EGC: enteric glial cell; EEC: enteroendocrine cell; LPS: lipopolysaccharide; SCFA: short-chain fatty acids.

Increasing evidence suggests a reciprocal relationship between gastroenterological and neurological manifestations in PD. This relationship begins in the prodromal phase of the disease when neurological manifestations can be very mild or absent. At this stage, in the “gut-brain” phenotype of PD, the disease can be recognised as an “intestinal syndrome”, where several factors such as microbial dysbiosis, leaky gut/endotoxemia, and intestinal inflammation, can lead to peripheral α-synucleinopathy and intestinal dysfunction (Figure 2). Recognising the presence of GI disorders in this phase could potentially enable the identification of people at risk of developing PD to offer them disease-modifying therapies before the central spread of pathological α-synuclein.

The connection between the GI tract and PD continues as the disease progresses, leading to a wide variety of gastroenterological phenomena that can cause severe disability, reduced quality of life and increased mortality. Some of the most common PD-related GI disorders also represent a major determinant of levodopa bioavailability and motor function. Prioritising the evaluation and treatment of these conditions is therefore a key factor in reducing the burden of motor fluctuations affecting individuals with PD. Therapeutic interventions through the manipulation of the gut-brain axis (e.g. use of dietary soluble fibres) have shown beneficial effects in the management of PD patients, both at the *gut* and *brain* level. The cooperation between different specialists (neurologists, gastroenterologists, internists and general practitioners), therefore, becomes essential in the clinical care of patients with PD.

## Data Availability

Data sharing is not applicable to this article as no new data were created or analysed in this study.
